# An examination of bedtime media and excessive screen time by Canadian preschoolers during the COVID-19 pandemic

**DOI:** 10.1186/s12887-022-03280-8

**Published:** 2022-04-18

**Authors:** C. Fitzpatrick, M. L Almeida, E. Harvey, G. Garon-Carrier, F. Berrigan, M Asbridge

**Affiliations:** 1 Département de l’enseignement au préscolaire et au primaire, Université de Sherbrooke, Sherbrooke, Canada; 2 Department of Childhood Education, University of Johannesburg, Gauteng, South Africa; 3 Federal University of Rio Grande Do Sul, Porto Alegre, Brasil; 4 Département des sciences de l’éducation, Université Sainte-Anne, Church Point, Canada; 5 Département de psychoéducation, Université de Sherbrooke, Sherbrooke, Canada; 6 Faculté des sciences de l’activité physique, Université de Sherbrooke, Sherbrooke, Canada; 7 Department of Community Health and Epidemiology, Dalhousie University, Halifax, Canada; 8 Department of Emergency Medicine, Dalhousie University, Halifax, Canada

**Keywords:** Preschooler, Early childhood education, Digital media use, Bedtime media, Screen time, Young children, Parental mediation, Temperament, Parental screen time

## Abstract

**Background:**

Risky media use in terms of accumulating too much time in front of screens and usage before bedtime in early childhood is linked to developmental delays, reduced sleep quality, and unhealthy media use in later childhood and adulthood. For this reason, we examine patterns of media use in pre-school children and the extent to which child and family characteristics contribute to media use during the COVID-19 pandemic.

**Methods:**

A cross-sectional study of digital media use by Canadian preschool-aged children (mean age = 3.45, *N* = 316) was conducted at the start of the COVID-19 pandemic between April and August of 2020. Parents completed a questionnaire and 24-h recall diary in the context of an ongoing study of child digital media use. From these responses we estimated hours of average daily screen time, screen time in the past 24 h, average daily mobile device use, and media use before bedtime. Parents also answered questions about their child (i.e., age, sex, temperament), family characteristics (parental mediation style, parental screen time, education, income), and contextual features of the pandemic (ex., remote work, shared childcare). Daycare closures were directly assessed using a government website.

**Results:**

Our results indicate that 64% of preschoolers used more than 2 h of digital media hours/day on average during the pandemic. A majority (56%) of children were also exposed to media within the hour before bedtime. Logistic and multinomial regressions revealed that child age and temperament, restrictive parental mediation, as well as parent digital media use, education, satisfaction with the division of childcare, remote work, and number of siblings and family income were all correlates of risky digital media use by preschoolers.

**Conclusions:**

Our results suggest widespread risky media use by preschoolers during the pandemic. Parenting practices that include using more restrictive mediation strategies may foster benefits in regulating young children’s screen time.

Concerns over the use of digital media by children have grown over the past decades. Early childhood represents a sensitive time in development because of the rapid pace of environment-dependent brain growth that occurs [1]. The development of brain networks that support learning and language depend on children experiencing high quality stimulation from their caregivers [2, 3]. Diets rich in digital media run the risk of displacing time for more enriching activities. Research indicates that digital media use between the ages of 2 and 5 is associated with developmental delays and later academic and social difficulties [4, 5]. Furthermore, research has linked unhealthy media habits in in 3- to 5-year-olds to reduced white matter integrity in brain areas that support language and the control of attention [6].

Over the past decade, the popularity of mobile devices (tablets, mobile phones) has been steadily increasing among children below the age of 8 [7]. Despite the rapid proliferation of digital media use, there has been limited research on use by young children [8]. The use of mobile devices is likely to differ from the use of more traditional media (i.e., watching television shows and movies), in several ways. Mobile devices are more interactive and provide sensory motor stimulation through the use of touchscreens. Indeed, research suggests that mobile devices are most frequently used to play games [9]. A recent study suggests that the use of mobile touch screen devices (i.e., smart phones, tablets) can compromise social development by age 5, by diminishing preschooler’s capacity to take the perspective of others, a competence known as theory of mind [10]. Given that mobile device use may be associated with specific risks to development, it remains important to better understand their use by young children.

In addition to fostering neuropsychological risk, sedentary behaviors developed in early childhood, including media use habits, are likely to be maintained through middle childhood and increase the risk of obesity [11, 12]. By adolescence, heavy screen media use (more than 3 h a day) is associated with obesity, and a reduction in healthy HDL cholesterol, and high blood pressure [13–15]. Furthermore, some research has found that two hours a day of screen time in adolescence is enough to have a negative effect on insulin levels [16]. Although Canadian child obesity and overweight rates have been stabilizing and decreasing, prior to the pandemic, they remained high with estimated prevalence of 13% and 27% for obesity and overweight, respectively [17]. As such, understanding and preventing heavy screen time use in early childhood is likely to have protective health effects up to adolescence.

Beyond excess screen time, the timing of media use is also of concern. The blue light emitted by screens can interfere with melatonin production and circadian rhythms [18, 19]. Research has specifically linked nighttime screen media use to decreased sleep quality and higher risk of neuropsychological problems such as reduced theory of mind [20].

Organizations preoccupied with the health and wellbeing of children recommend that preschool-aged children between the ages of 2 and 5 be exposed to no more than 1 h of digital media per day. Furthermore, it is recommended that children not be exposed to digital media the hour before bedtime [21–23]. Prior to the pandemic, many preschool children exceeded the recommended healthy allowance of 1 h a day of screen time for 3–5-year-olds while bedtime usage rates are less well known [24]. Since the start of the COVID-19 pandemic in the winter of 2020, public health related restrictions have significantly reduced opportunities for real life social, educational, and leisure activities. According to one study, this has resulted in estimated increases in digital media exposure among children and young people 18 years and younger [25].

The individual child and family characteristics which contribute to patterns of media use, remain largely unexamined. Child sex and age are likely to impact exposure to media. Research suggests that older children spend more time with digital media [26]. Furthermore, boys spend more time in front of screens than girls [27, 28]. Family distress hypotheses suggest that parents expose children to more media when facing higher levels of family adversity. In line with this hypothesis, research also suggests that preschoolers with challenging temperamental characteristics in terms of extraversion and negative affectivity are exposed to more screens use [29–31]. This may lead to a transactional process by which fussier toddlers, due to greater media exposure, receive reduced amounts of time for socially enriching activities, further contributing to poor self-regulation, and in turn more media exposure.

Parental mediation of their child’s digital media use may also contribute to young children’s digital media habits. Previous work has identified three types of strategies: 1) Restrictive mediation, which involves setting rules and limits on child media activities; 2) Instructive or active mediation which involves discussing the content of media with children; and 3) Social co-viewing, which involves the shared viewing of media without discussing its content [32]. There is some evidence that less educated, lower income families, and parents with less confidence in their digital media skills are more likely to use restrictive types of mediation [27]. Research with school-aged children suggests that restrictive mediation is most effective in reducing digital media exposure [33]. Co-viewing has also received attention as a promising strategy for maximizing the positive outcomes of media use and reducing its potential risks [34], while instructive mediation has been linked to improved learning from educational media [35].

Related to mediation style, parents own media use is also likely to influence child habits. The interference of technology in parent–child relationships, a phenomenon known as technoference, has been linked to reductions in the frequency and quality of family interactions [36]. Research also suggests that the amount of time parents spend with digital media is associated with media use in 5 and 6-year-olds [37]. The extend to which parental media use may contribute to preschool children’s media habits has been less examined. Finally, the larger social context in which families find themselves is also likely to influence child media use. Children in more disadvantaged families are likely to spend more time with screens, which may then result in more vulnerabilities in their school readiness [38]. Futhermore, research has found that COVID-19 related changes in the availability of childcare and family resources are related to increases in family distress and media use [25].

The objective of the present study is to better understand young children’s digital media use during the COVID-19 crisis. We aim to describe patterns of risky media use that include spending upwards of 2 h/day with digital media and media use before bedtime. We also examine how child (i.e., age, sex, temperament) and family characteristics (parental mediation style, parental screen time, education, income) and contextual features of the pandemic (ex., remote work, daycare closure, shared childcare) are associated with media use by children. Our hypothesis is that more family distress (i.e., more challenging child temperaments, lower income) will be associated with less healthy preschool media habits.

## Methods

### Sample

This study draws on participants engaged in a larger two-year longitudinal study of Canadian parents with children between the ages of 2 and 5 (mean age 3.46) who completed an assessment of child digital media use between April and August of 2020 during the first wave of the COVID-19 pandemic (*N* = 316 children). Participants were recruited by distributing eye catching posters and flyers to preschools and pre-kindergarten classes, through sign up sheets and presentations given at preschool and pre-kindergarten registration nights, a Facebook page, and newspaper and radio advertisements broadcast across Nova Scotia Canada. Data was collected during a provincially declared state of emergency and lockdown. For 20.1% (*N* = 64) of our sample, daycares were closed at the time of data collection, while the remaining 79.1% (*N* = 254) completed the assessments after daycares could reopen. Mothers were the primary respondent (*N* = 295 or 93.4%). Most respondents reported being married (82%), born in Canada (91%), and white (90.5%). Our sample contained slightly more boys (*N* = 168) than girls (*N* = 146). Finally, our sample was predominantly English speaking with 88.1% (*N* = 280), reporting that English is the primary language spoken in their home.

### Data collection procedure

Parents completed the web-based CAFÉ assessment of family digital media use. This assessment has been described elsewhere [8]. This web-based assessment of family media exposure included a survey and 24-h recall diary. The survey includes questions on child age and sex and parent education and income, as well as items measuring child and parent media use and parental mediation of child screen use. For our study, we integrated questions on child temperament, as well as contextual questions relating to the COVID-19 crisis, including changes in family work situation, and satisfaction with the division childcare within the household. Information on child screen time was also collected using the 24-h recall diary. These measures are described below.

### Measures: Outcomes

Child average daily screen time. Parents completed the Media assessment Questionnaire (MAQ) [8] in which they reported the average amount of time children spent doing each of the following on weekdays and weekend days separately: (1) watching TV or DVDs; (2) using a computer; (3) playing video games on a console; (4); Using an iPad, tablet, LeapPad, iTouch, or similar mobile device (excluding smartphones); or (5) Using a smartphone. Response options included: (1) Never; (2) Less than 30 min; (3) 30 min to 1 h; (4) 1–2 h; (5) 2–3 h; (6) 4–5 h; (7) more than 5 h. We then converted these categorical responses into a variable reflecting the number of hours spent with each type of media. Our approach involved using the midpoint for each response range, with the exception of “5 or more hours a day” where a more conservative score of 5 was used*.* Weekly estimates were then estimated by multiplying weekday estimates by 5 and weekend day estimates by 2 and dividing the total by 7. Finally, we calculated an overall daily screen time estimate by summing average daily usage across media devices.

Mobile device use. A separate score was computed for average daily mobile device use based on the summed amount of time spent using tablets and smartphones. Daily estimates of mobile device use were then dichotomized to reflect either (0) being exposed to an average of 2 h or less of mobile device use or (1) exposed to more than 2 h/day of average mobile device use.

Screen time was also measured using a 24-h time use diary [8]. This measure allowed parents to log the amount of time their child spent: Sleeping/resting; Eating/drinking; Bathing/personal grooming; Play/recreation inside; Play/ recreation outside; Childcare; Out of the house (travelling/errands); In house/doing chores; or doing “Other activities”. Parents entered the time and duration of media use using 15 min intervals. To capture heavy exposure to digital media we used a similar approach as with the questionnaire data and categorised children as either (0) exposed to 2 h or less of media during the previous day or (1) exposed to more than 2 h of media the previous day.

Device use before bedtime. Parents also reported how often their child used the following media the hour before bedtime: (1) Television, (2) DVR; (3) DVD or VCR; (4) Personal computer; (5) Smartphone; (6) Ipad or tablet; (7) Console based gaming system; (8) Video streaming service. Responses were coded as: (1) Never; (2) less than once a week; (3) About once a week; (4) 2–3 times a week; (6) 4–6 times a week; (7) Every night. Based on these responses, participants were categorised as either: (1) Never or rarely exposed (once a week or less), (2) Moderately exposed (between 1 and 3 nights/week), Habitually exposed (4–7 nights a week).

### Measures: Child and family predictors

Child characteristics. Child age, sex, and temperament were reported by parents. Temperament was measured using the Children’s Behavior Questionnaire—Short Form [39]. This instrument measures distinct dimensions of temperament that can be grouped into three factors: Negative affectivity, surgency/extraversion, and effortful control. Negative affectivity was based on scores on dimensions of anger/frustration and falling reactivity, surgency/extraversion was based on scores on the dimensions of shyness and impulsivity and finally, effortful control was based on scores on the dimensions of attentional focusing and inhibitory control. Higher score on negative affectivity indicates greater intensity and duration of the child’s response to environmental stimuli, while surgency/extraversion indicate higher levels of impulsivity and activity. The third factor, effortful control, refers to the child’s abilities to self-regulate their level of reactivity. The short version uses a 7-point Likert scale ranging from 1 (*extremely untrue of your child*) to 7 (*extremely true of your child*). The internal consistency coefficients were 0.84 for negative affectivity, 0.84 for surgency/extraversion, and 0.79 for effortful control.

Family characteristics. Parents completed the Valkenburg scale [32] to measure parent strategies for monitoring their child’s media use. Parents indicated their agreement with the following statements on a Likert scale with response options: 1 (never); 2 (rarely); 3 (sometimes); and 4 (often). The following items were used to measure: *Social coviewing* (Watch together because you both like the program; Laugh with the child about the things you see on TV; Watch together because of a common interest in a program, alpha = 0.85); *Instructive mediation* (Try to help the child understand what s/he sees on TV; Point out why some things actors do are bad; Explain what something on TV really means; Explain the motives of TV characters; Point out why some things actors do are good, alpha = 0.86); *Restrictive mediation* (Set specific viewing hours for your child; Restrict the amount of child viewing; Tell your child to turn off the TV when they are watching an unsuitable program; Tell your child in advance the programs they may watch; Forbid your child to watch certain programs, alpha = 0.64).

Parents also reported how often they personally engaged in the following activities at home on a typical weekday or weekend day separately: (1) Watching TV or DVDs; (2) Using a computer; (3) Playing video games on a console; (4); Using an iPad, tablet, LeapPad, iTouch, or similar mobile device (excluding smartphones); or (5) Using a smartphone (not including talking on the phone). Response options included: (1) Never; (2) Less than 30 min; (3) 30 min to 1 h; (4) 1–2 h; (5) 2–3 h; (6) 4–5 h; (7) more than 5 h. Scores were converted into measures of average digital media use per day by multiplying weekday estimates by 5 and weekend day estimates by 2 and dividing the total by 7.

Parents reported levels of education, income, satisfaction with the division of childcare, and changing to remote work. Education reflects the highest school grade completed by the parent. Responses were categorized as either: (1) High school or college vocational; (2) Undergraduate; or (3) Graduate degree. Household income was categorized as either (1) less than 60,000 CND; (2) 60,000–99 000 CND; or (3) 100 000 CND or higher. Parents also reported the number of children in their household. Satisfaction with childcare was assessed with the following question: How satisfied are you with the division of childcare between you and your partner? Responses were recorded as: (1) Very satisfied; (2) Satisfied; (3) Not satisfied or unsatisfied; (4) Unsatisfied; (5) Very unsatisfied. Answers were recoded to create a dichotomous variable reflecting (1) Being unsatisfied or neither satisfied or unsatisfied or (0) indicating being satisfied or very satisfied. Finally, responding parents indicated COVID-related changes in their work situation by selecting from the following options: (1) You/you’re partner are now working from home; your/your partner’s work hours were reduced; your/your partner’s work hours have increased; your/your partner’s on unpaid leave; you/ your partner recently got laid off; You’ve/your partner’s stopped working to take care for your child/children; Your/your partners work situation has remained the same; other. Responses were scored as 1 if the parent indicated they or their partner were now working from home or 0 if they selected any other response. Finally, daycare closures were directly inferred based on the dates that daycares were ordered to close and eventually allowed to reopen (novascotia.ca/news/release/?id = 20,200,313,009). Closure was scored as 0 and open as 1.

### Data analytic strategy

We first describe patterns of child media use and individual and family characteristics of our sample. Then we examine the extent to which these characteristics contribute to the risk of unhealthy media habits. In our sample, only a minority of children (*N* = 46, 15%), respected the recommended 1 h or less per day [21]. As such, to better reflect the high levels of media use in our sample, we categorized children as either (0) exposed to an average of 2 h or less of daily media or (1) exposed to more than 2 of daily media per day on average. Multivariable logistic regressions are used to model associations between child and family characteristics and the probability of accumulating 2 h or more of average daily total digital media use and mobile device use. We also estimate odds of exceeding 2 h of digital media in the past 24-h. Finally, we estimate the probability of being never/rarely (less than 1/week), moderately, or habitually exposed to media before bedtime. Associations between child and family characteristics and media use before bedtime is estimated using multinomial logistic regression to identify characteristics that distinguish between children who were habitually exposed to media before bedtime from those who were rarely/never exposed or moderately exposed.

## Results

### Descriptive results

Descriptive statistics and frequencies for categorical variables are presented in Tables 1 and 2. In total, 63.8% of our sample was exposed to more than two hours of digital media per day on average and 23.2% of our sample was exposed to two hours of mobile devices use per day on average. When asked to report exposure during the last 24-h, only 36% of families reported more than two hours of exposure. Finally, in terms of media exposure before bedtime, most of our sample was exposed habitually (56%) as compared to 27% exposed moderately and 17% exposed never/rarely.

**Table 1:** Descriptive statistics for continuous child, parent, and family variables

	Mean 95% CI	SD	Min–Max	N (% missing)
*Child characteristics*				
Age	3.46 (3.36–3.55)	.86	2.00–5.42	316 (0)
Effortful control	4.70 (4.61–4.80)	.85	2.50–7.00	315 (0.3)
Negative affectivity	3.61 (3.51–3.71)	.90	1.00–6.92	315 (0.3)
Extraversion	4.27 (4.17–4.38)	.98	2.00–6.58	315 (0.3)
*Family characteristics*				
Coviewing	3.09 (3.02–3.16)	.63	1.00–4.00	311 (2.53)
Restriction	3.18 (3.10–3.25)	.68	1.00–4.00	311 (1.58)
Instructive	3.13 (3.06–3.20)	.65	1.40–4.00	308 (1.58)
Parent screen time (hours/day)	6.34 (6.01–6.69)	3.07	0.75–14.94	316 (0)
Number of children	2.00 (1.91–2.09)	.83	1.00–5.00	316 (0)

**Table 2:** Frequency distributions for categorical variable

	%	n	Total N (% missing)
*Child media use*			
Screen time (MAQ)			315 (0.3)
0–2 h/day	36.2	114	
> 2 h/day	63.8	201	
Mobile device use (MAQ)			315 (0.3)
0–2 h/day	76.8	242	
> 2 h/day	23.2	73	
Screen time (time use diary)			239 (24)
0–2 h/day	64	153	
> 2 h/day	36	86	
Media use before bed			315 (0.3)
Never or rarely (< 1 time/week)	17.5	55	
Moderate (1–3 time/week)	26.7	84	
Habitual (4–7 time/week)	55.9	176	
*Child characteristics*			
Sex			316 (0)
Boys	169	53.5	
Girls	147	46.5	
*Family characteristics*			
Division of childcare			305 (3)
Satisfied	71.1	217	
Unsatisfied	28.9	88	
Parent education			316 (0)
HS/vocational	25.6	81	
Bachelors	46.2	146	
Graduate	28.2	89	
Family income			296 (6)
< 60,0000	15.9	47	
600,000–100,000	29.4	87	
> 100,000	54.7	162	
Remote work (responding parent)			315 (0.3)
No	68.4	216	
Yes	31.4	99	
Remote work (partner)			316 (0)
No	70.9	224	
Yes	29.1	92	
Daycare			316 (0)
Open	79.7	252	
Closed	20.3	60.4	

### Missing data

The proportion of missing data on outcome variable ranged from 0.03 to 24%. In order to reduce the effect of bias due sample attrition and to maintain statistical power [40], we conducted multiple imputations using SPSS. Results of regression analyses therefore represent pooled estimates over 5 estimated imputed data sets.

### Logistic regression

Adjusted logistic and multinomial regression models are presented in Tables 3 and 4 respectively. Across all three models, child age contributed significantly to media use. More specifically, each year increase in child age was associated with a 205% higher likelihood of exceeding two hours of average daily screen time (odds ratio = 2.05, 95% confidence interval [CI], 1.44 to 2.93). Older children were exposed to more mobile devices with each year increase in age corresponding to a 159% increase in the odds of exceeding two hours of average daily mobile device use (odds ratio = 1.59, 95% CI, 1.11 to 2.80), and a 197% increase in the odds of exceeding two hours of screen time in the last 24 hrs (odds ratio = 1.97, 95% CI, 1.40 to 2.80).

**Table 3:** Adjusted logistic regression estimating the contribution of child and family characteristics to excessive screen time

	Average daily child ST (reference = less than 2 h/day)		Average daily mobile device use (reference = less than 2 h/day)		Child ST last 24 hrs (reference = less than 2 h/day)	
	Odds ratio (95% CI)	*P*-Value	Odds ratio (95% CI)	*P*-Value	Odds ratio (95% CI)	*P*-Value
Child age (years)	**2.05 (1.44–2.93)**	** < .0001**	**1.59 (1.11–2.29)**	**.011**	**1.97 (1.40–2.80)**	** < .0001**
Child sex						
Girl	.74 (.42–1.31)	.297	.75 (.41–1.39)	.367	.80 (.37–1.74)	.560
Boy (reference)	–-	–-	–-	–-	–-	–-
*Child temperament*						
Effortful control	1.04 (.69–1.58)	.915	.86 (.54–1.36)	.508	.90 (.55–1.45)	.642
Negative affectivity	1.20 (.82–1.74)	.350	1.21 (.82–1.81)	.339	.89 (.60–1.34)	.580
Extraversion	1.28 (.94–1.73)	.115	.98 (.71–1.34)	.883	.95 (.61–1.48)	.815
*Parental mediation*						
Coviewing	1.65 (.99–2.76)	.056	1.22 (.69–2.16)	.485	1.10 (.66–1.82)	.714
Restrictive	**.46 (.29-.75)**	**.002**	**.43 (.26-.69)**	** < .0001**	**.60 (.39-.92)**	**.020**
Instructive	.81 (.49–1.34)	.409	1.69 (.97–2.94)	.066	.99 (.48–2.04)	.985
Parent ST (hours)	**1.29 (1.15–1.46)**	** < .0001**	1.08 (.98–1.19)	.129	1.04 (.93–1.17)	.508
Division of childcare						
Unsatisfied	1.74 (.89–3.4)	.106	1.37 (.66–2.87)	.397	**2.48 (1.15–5.34)**	**.022**
Satisfied (ref)	––	–-	–-	–-	–-	–-
Parent education						
HS/vocational	1.46 (.62–3.46)	.389	1.52 (.63–3.68)	.350	**3.19 (1.03–9.94)**	**.046**
Bachelors	1.50 (.77–2.94)	.234	.93 (.42–2.06)	.858	1.97 (.80–4.85)	.133
Graduate (ref)	–-	–-	–-	–-	–-	–-
Family income						
< 60,0000 $	2.43 (.87–6.80)	.092	**3.27 (1.32–8.09)**	**.010**	1.46 (.45–4.75)	.509
60,000–100,000 $	1.14 (.58–2.25)	.706	1.07 (.49–2.36)	.860	.73 (.37–1.46)	.368
> 100,000 $ (ref)	–-	–-	–-	–-	–-	
Number of children	.75 (.53–1.07)	.108	.77 (.53–1.13)	.177	1.14 (.77–1.68)	.511
Remote work parent						
No	**2.73 (1.33–5.60)**	**.006**	1.29 (.60–2.78)	.508	.87 (.45–1.69)	.672
Yes (ref)	–-	––	–-	–-	–-	–-
Remote work partner						
No	.82 (.42–1.60)	.563	.99 (.48–2.05)	.977	.96 (.41–2.22)	.913
Yes (ref)	–-	–-	–-	–-	–-	–-
Daycare						
Closed	1.18 (.58–2.41)	.645	1.39 (.66–2.92)	.385	1.14 (.55–2.37)	.714
Open (ref)	–-	–-	–-	–-	–-	–-

*ST* Screen time hours

**Table 4:** Multinomial logistic regression examining the contribution of child and family characteristics to media use before bedtime

	Media use before bedtime (Reference = Habitually exposed /4–7 times/week)			
	Never/rarely exposed (less than 1/week)		Moderately exposed (1 to 3 times/week)	
	Odds ratio (95% CI)	*P*-Value	Odds ratio (95% CI)	*P*-Value
Child age	.71 (.44–1.12)	.141	.81 (.57–1.15)	.232
Child sex				
Girls	.97 (.47–2.03)	.940	1.46 (.82–2.61)	.203
Boys (reference)	–-	–-	–-	–-
*Child Temperament*				
Effortful control	**2.01 (1.17–3.45)**	**.012**	**1.56 (1.01–2.41)**	**.045**
Negative affectivity	.99 (.61–1.60)	.961	.88 (.60–1.29)	.517
Extraversion	.82 (.55–1.22)	.337	1.15 (.84–1.56)	.383
*Parental mediation style*				
Coviewing	**.24 (.12-.47)**	** < .0001**	1.11 (.64–1.92)	.721
Restrictive	**2.57 (1.37–4.79)**	**.003**	**1.81 (1.12–2.91)**	**.015**
Instructive	1.23 (.64–2.36)	.527	**.51 (.30-.85)**	**.010**
Parental ST (hours)	.91 (.79–1.04)	.167	**.86 (.77-.96)**	**.009**
*Division of childcare*				
Satisfied	1.59 (.68–3.64)	.288	.74 (.39–1.40)	.347
Unsatisfied (ref)	–-	–-	–-	–-
Parent education				
HS/vocational	.60 (.19–1.90)	.385	1.06 (.43–2.66)	.896
Bachelors	.99 (.43–2.31)	.988	1.66 (.80–3.42)	.172
Graduate (ref)	–-	–-	–-	–-
Family income				
< 60,0000 $	.52 (.14–1.95)	.330	.53 (.20–1.43)	.210
60,000–100,000 $	.61 (.25–1.49)	.276	.97 (.49–1.93)	.926
> 100,000 $ (ref)	–-	–-	–-	–-
Number of children	**1.69 (1.07–2.65)**	**.024**	1.00 (.70–1.42)	.989
Remote work				
Yes	1.85 (.76–4.55)	.178	1.98 (.99–3.98)	.054
No (ref)	–-		–-	–-
Remote work partner				
Yes	1.03 (.45–2.36)	.938	1.15 (.59–2.23)	.688
No (ref)	–-	–-	–-	–-
Daycare				
Closed	1.01(.40–2.58)	.981	.80 (.38–1.69)	.558
Open (ref)	–-	–-	–-	–-

*ST* Screen time hours

Parental mediation style was also significantly related to child media habits. The use of restrictive mediation by parents was associated with a 54% reduction in the odds of exceeding two hours of average daily screen time (odds ratio = 0.46, 95% CI, 0.29-0.75), a 57% reduction in the odds of exceeding an average of two hours of mobile device use (odds ratio = 0.43, 95% CI, 0.26 to 0.69), and a 40% reduction in the chances of exceeding two hours of screen time in the last 24 hrs (odds ratio = 0.60, 95% CI, 0.39 to 0.92). A greater use of instructive mediation predicted 184% increase in the odds of exceeding an average of two hours of daily mobile device use (odds ratio = 1.84, 95% CI, 1.06 to 3.21). Parental co-viewing did not predict child media use. Parents own screen time contributed to child habits with each hour of parental daily screen time contributing to a 129% increase in the odds of children exceeding two hours of average daily screen time (odds ratio = 1.29, 95% CI, 1.15–1.46). Lower parental satisfaction with the division of childcare was also associated with a 248% increase in the odds of children exceeding two hours of average daily media use in the last 24 hrs (odds ratio = 2.48, 95% CI, 1.15–5.34). Parental education and income also contributed to child media habits. Parents with a high school or vocational degree were 319% more likely to have exposed their children to two hours or more of screen time in the last 24 hrs (odds ratio = 3.19, 95% CI, 1.03 to 9.94) when compared to parents with a graduate degree. Parents who made less than 60,000$ a year were 327% more likely to expose children to two hours or more of screen time in the last 24 hrs (odds ratio = 3.27, 95% CI, 1.32 to 8.09. Finally, parents who did not transition to working from home were 273% more likely to expose children to more than two hours of average daily screen time (odds ratio = 2.73, 95% CI, 1.33–5.60).

### Multinomial regression

In terms of media use before bedtime, multinomial logistic regression was used to compare children that were habitually exposed (4–7 times/week) to children that were never/rarely exposed (less than 1/week) and moderately exposed (1–3 times/week). A one-point increase in regulation abilities (effortful control) was associated with a 201% increase in the odds of being never/rarely vs habitually exposed (odds ratio = 2.01, 95% CI, 1.17–3.45) and was associated with a 156% increase in the odds of being exposed regularly vs frequently (odds = 1.56, 95% CI, 1.01–2.41). Parental co-viewing was associated with a 76% reduction in the odds of being never/rarely vs habitually exposed (odds ratio = 0.24, 95% CI, 0.12 to 0.47) whereas restrictive mediation was associated with a 257% increase in the odds of being never/rarely exposed vs habitually exposed (odds ratio = 2.57, 95% CI, 1.37–4.79) and was associated with a 181% increase in the odds of being moderately vs habitually exposed (odds ratio = 1.81, 95% CI, 1.12–2.91). Instructive media also decreased the probability of being moderately vs habitually exposed by 49% (odds ratio = 0.51, 95% CI, 0.30 to 0.85). Finally, each additional hour of a parent own screen time also decreased the odds of being exposed moderately vs habitually by 14% (odds ratio = 0.86, 95% CI, 0.77 to 0.96).

## Discussion

The present study aimed to better understand patterns of media use by young Canadian children during the pandemic and to identify characteristics of children and their families associated with more risky patterns of digital media use, such as spending upwards of 2 h a day with digital media and using media before bedtime. Most children (64%) were exposed to twice the amount of average daily screen time recommended for preschool aged-children and were frequently exposed to bedtime media. When using a 24-h diary estimates were lower, with less than half (36%) of children exceeding two hours of screen time. Furthermore, in our sample, fewer children (23%) exceeded more than 2 h per day of tablets and smartphones use, indicating that viewing television shows and movies remains the most frequent digital media use activity among children [26].

In terms of individual correlates of heavy digital media use, older children were more likely to be exposed to more than 2 h/day of screen time and mobile devise use than younger children. Parents own media use also predicted a greater risk of children exceeding two hours of media daily. In contrast, parents who adopted a restrictive mediation style, in terms of setting time and content limits, were less likely to expose their child to heavy screen time and mobile devise use. In terms of demographic and COVID-related contextual variables, parents who worked from home were less likely to have children who exceeded two hours of media per day. More educated parents were less likely to report that their child exceeded two hours of media in the past 24 hrs and parents with higher income and who were more satisfied with the division of childcare were also less likely to have children exceeding 2 h of average mobile device use.

Child and family characteristics also contributed to the risk of being exposed to media before bedtime. Children who had better temperamental effortful control, meaning who were better able to regulate their emotions, attention, and movement, were less likely to be exposed to media before bedtime. Parents using a restrictive style also exposed their children to bedtime media less frequently. In contrast, co-viewing and instructive parents exposed their children to bedtime media more often. Parents who used more media themselves, were also more likely to use bedtime media with children. Finally, parents who reported having more children, used less bedtime media.

Our results provide partial support for family distress models of child digital media use [25]. In particular, lower parental education, income, and satisfaction with the division of childcare, as well as more challenging child temperaments were all associated with worse preschool media habits. Surprisingly, however child sex, number of siblings, remote work, and daycare closures were not associated with unhealthy preschooler media habits.

The present findings can be interpreted within the context of previous research. Prior to the pandemic, two Canadian studies indicated that between 46 and 58% of preschool aged children respected the recommendation of < 1 h/day of screen media [41, 42]. In comparison, a similar proportion (63%) of preschoolers in our study were exposed to more than 2 h of digital media daily, suggesting increases in media use among preschoolers during the pandemic. Our findings with regards to correlates of preschooler media use are also similar to those of another study of Canadian 3-year-olds, conducted prior to the pandemic. More specifically, others have found that parental screen use was a significant correlate of child screen media use, whereas child sex was unrelated to child time spent with media [43]. Unlike our study, however, Madigan et al. did not find any associations between family demographic characteristics and child screen time.

In our study, parental mediation of children’s screen time was a consistent correlate of child media habits. A study conducted with Hungarian children between the ages of 0 and 7 found that parents with a lower educational level and who were more permissive, had children who spent more time using mobile devices [9]. Furthermore, there is qualitative research suggesting that older school-aged children are likely to perceive co-viewing as a parental endorsement of screen media use. Whereas restrictive mediation is more likely to be interpreted as parental disapproval of media [44]. The extent to which this may apply to preschool age children remains to be examined.

The present results need to be interpreted within certain limitations. First, the present study is based on a single wave of a larger longitudinal study, which precludes us from determining the direction of association. For instance, it may be the case that parental mediation exercises an influence on child media habits. However, it may also be the case that children with greater or lesser appetites for media illicit different parental strategies. Another limit of our study is the use of a relatively homogenous, low risk convenience sample. As such, our findings may not be generalizable to the population of Canadian preschoolers. It is also possible that indicators of family distress (remote work, daycare closures) may have had a greater impact on child media habits in more diverse or at-risk samples. Finally, in our study, parents only provided 24 h recall based on one weekday. Other studies recommend that estimates include data from two non-consecutive days [45]. In terms of strengths, the present study is enhanced by using multiple measures of child screen time habits and the availability of detailed measures of child and family characteristics. Finally, to our knowledge there remains limited research on young children’s media habits during the COVID-19 pandemic.

When activities are hard or impossible to avoid, as is the case with screen time, theory and research support the effectiveness of harm reduction approaches that focus on empowering individuals to make changes to their behaviors to minimize risks to their health and wellbeing [46]. As such, our results indicate that parental mediation may be a promising target for clinical interventions and public health campaigns that aim to promote healthy media habits starting from a young age. Beyond restricting screen time, additional strategies are likely to also be effective in helping parents regulate child screen time. According to a qualitative study effective parental strategy may involve setting rules based on timing of use (no media before bedtime or during meals), collaborative rule setting, developing a family bank of activities that do not involve media, and helping children learn to regulate their own media use [47]. The extent to which these different strategies are predictive of child media habits warrants investigation by longitudinal studies.

Future research, including forthcoming work with subsequent waves of our study will help clarify the extent to which child temperament and parental monitoring strategies prospectively contribute to child media habits. As well, future studies should address the extend to which child media use during the pandemic contributes to social and developmental outcomes. For instance, bedtime media has been linked to decreased sleep quality and eventual reduction in effortful control [48]. However, a reverse association is also possible whereby children with better effortful control illicit fewer parental resources which in turn decreases their exposure to bedtime media [25]. Longitudinal investigations will be useful in clarifying the direction of these effects.

## Conclusion

Our results indicate that unhealthy patterns of media use are widespread among preschoolers and suggest that many children are failing to meet the healthy screen time recommendations from the World Health Organisation, the American Academy of Pediatrics, the Canadian Association for exercise physiology and the American Academy of Child and Adolescent Psychiatry during the COVID-19 pandemic [21–23]. Poorly regulated early childhood media use habits can pave the way of poor media habits in later childhood and adolescence as well as poor health, school readiness, and wellbeing. For this reason, it remains important to develop tailored, family-based interventions that can be implemented as early as the preschool years.

## Data Availability

The data that support the findings of this study are available upon request.

## References

[1] Shonkoff JP (2016). Capitalizing on advances in science to reduce the health consequences of early childhood adversity. JAMA Pediatr.

[2] Rohr CS, Arora A, Cho IYK (2018). Functional network integration and attention skills in young children. Dev Cogn Neurosci.

[3] Sousa SS, Amaro E, Crego A, Gonçalves ÓF, Sampaio A (2018). Developmental trajectory of the prefrontal cortex: a systematic review of diffusion tensor imaging studies. Brain Image Beh.

[4] Madigan S, Browne D, Racine N, Mori C, Tough S (2019). Association between screen time and children’s performance on a developmental screening test. JAMA Pediatr.

[5] Pagani LS, Lévesque-Seck F, Fitzpatrick C (2016). Prospective associations between televiewing in toddlerhood and later self-reported social impairment. Psych Med.

[6] Hutton JS, Dudley J, Horowitz-Kraus T, DeWitt T, Holland SK (2020). Associations between screen-based media use and brain white matter integrity in preschool-aged children. JAMA Pediatr.

[7] Rideout V (2017). The Common Sense census: Media use by kids age zero to eight.

[8] Barr R, Kirkorian H, Radesky J, Coyne S, Nichols D, Blanchfield O (2020). Beyond Screen Time: A synergistic approach to a more comprehensive assessment of family media exposure during early childhood. Frontiers Psych.

[9] Konok V, Bunford N, Miklósi Á (2020). Associations between child mobile use and digital parenting style in Hungarian families. J Children Media.

[10] Konok V, Liszkai-Peres K, Bunford N, Ferdinandy B, Jurányi Z, Ujfalussy DJ (2021). Mobile use induces local attentional precedence and is associated with limited socio-cognitive skills in preschoolers. Comp Hum Beh.

[11] Jones RA, Hinkley T, Okely AD, Salmon J (2013). Tracking physical activity and sedentary behavior in childhood: a systematic review. Am J Prev Med.

[12] Carson V, Tremblay MS, Chaput JP, Chastin SF (2016). Associations between sleep duration, sedentary time, physical activity, and health indicators among Canadian children and youth using compositional analyses. App Physiol Nut Metab.

[13] Merghani A, Malhotra A, Sharman S (2015). The U-shaped relationship between exercise and cardiac morbidity. Trends Cardiovasc Med.

[14] Goldfield GS, Kenny GP, Hadjiyannakis S, Phillips P, Alberga AS, Saunders TJ (2011). Video game playing is independently associated with blood pressure and lipids in overweight and obese adolescents. PLoS ONE.

[15] Martinez-Gomez D, Tucker J, Heelan KA, Welk GJ, Eisenmann JC (2009). Associations between sedentary behavior and blood pressure in young children. Arc Pediatric Adol Med.

[16] Hardy LL, Denney-Wilson E, Thrift AP, Okely AD, Baur LA (2010). Screen time and metabolic risk factors among adolescents. Arch Pediatric Adol Med.

[17] Rodd C, Sharma AK (2016). Recent trends in the prevalence of overweight and obesity among Canadian children. CMAJ.

[18] Cheung CHM, Bedford R, Saez De Urabain IR, Karmiloff-Smith A, Smith TJ. Daily touchscreen use in infants and toddlers is associated with reduced sleep and delayed sleep onset. Scientific Reports. 2017. 10.1038/srep46104. 10.1038/srep46104 PMC5390665 28406474

[19] Falbe J, Davison KK, Franckle RL, Ganter C, Gortmaker SL, Smith L (2015). Sleep duration, restfulness, and screens in the sleep environment. Pediatrics.

[20] Nathanson AI, Fries PT (2014). Television exposure, sleep time, and neuropsychological function among preschoolers. Media Psychol.

[21] American Academy of Child and Adolescent Psychiatry. Screen Time and Children. https://www.aacap.org/AACAP/Families_and_Youth/Facts_for_Families/FFF-Guide/Children-And-Watching-TV-054.aspx. Accessed 15 Sep 2021.

[22] Digital Health Task Force, Canadian Paediatric Society (2017). Screen time and young children: Promoting health and development in a digital world.

[23] World Health Organization. Guidelines on physical activity, sedentary behaviour and sleep for children under 5 years of age. Geneva: World Health Organization; 2019. Licence: CC BY-NC-SA 3.0 IGO. 31091057

[24] American Academy of Pediatrics (2020). What do we really know about kids and screens?.

[25] Hartshorne JK, Huang YT, Paredes PML, Oppenheimer K, Robbins PT, Velasco MD (2021). Screen time as an index of family distress. Cur Res Beh Sci.

[26] Rideout V, Robb MB (2020). The Common Sense census: Media use by kids age zero to eight, 2020.

[27] Blum-Ross A, Livingstone S (2016). Families and screen time: Current advice and emerging research. Media Policy Brief 17.

[28] Sanders W, Parent J, Forehand R, Sullivan AD, Jones DJ (2016). Parental perceptions of technology and technology-focused parenting: Associations with youth screen time. Journal of Applied Dev Psych.

[29] McArthur BA, Hentges R, Christakis DA, McDonald S, Tough S, Madigan S (2021). Cumulative social risk and child screen use: the role of child temperament. J Pediatric Psych.

[30] Thompson AL, Adair LS, Bentley ME (2013). Maternal characteristics and perception of temperament associated with infant TV exposure. Pediatrics.

[31] Radesky JS, Silverstein M, Zuckerman B, Christakis DA (2014). Infant self-regulation and early childhood media exposure. Pediatrics.

[32] Valkenburg PM, Krcmar M, Peeters AL, Marseille NM (1999). Developing a scale to assess three styles of television mediation:“Instructive mediation”,“restrictive mediation”, and “social coviewing”. J Broadcasting Electronic Media.

[33] Nathanson AI (1999). Identifying and explaining the relationship between parental mediation and children's aggression. Com Res.

[34] American Academy of Pediatrics (2013). Policy statement on children, adolescents, and the media. Pediatrics.

[35] Strouse GA, O'Doherty K, Troseth GL (2013). Effective coviewing: Preschoolers’ learning from video after a dialogic questioning intervention. Dev Psych.

[36] McDaniel BT, Radesky JS (2018). Technoference: Parent distraction with technology and associations with child behavior problems. Child Dev.

[37] Jago R, Thompson JL, Sebire SJ, Wood L, Pool L, Zahra J (2014). Cross-sectional associations between the screen-time of parents and young children: differences by parent and child gender and day of the week. Int J Beh Nut Phys Act.

[38] Ribner A, Fitzpatrick C, Blair C (2017). Family socioeconomic status moderates effects of television viewing and school readiness skills. J Dev Beh Ped.

[39] Putnam SP, Rothbart MK (2006). Development of short and very short forms of the Children's Behavior Questionnaire. J Pers Assess.

[40] Cummings P (2013). Missing data and multiple imputation. JAMA Pediatr.

[41] Carson V, Tremblay MS, Spence JC, Timmons BW, Janssen I (2013). The Canadian sedentary behaviour guidelines for the early years (zero to four years of age) and screen time among children from Kingston. Ontario Paediatrics Child Health.

[42] Tamana SK, Ezeugwu V, Chikuma J, Lefebvre DL, Azad MB, Moraes TJ, Subbarao P, Becker AB, Turvey SE, Sears MR, Dick BD (2019). Screen-time is associated with inattention problems in preschoolers: Results from the CHILD birth cohort study. PloS one.

[43] Madigan S, Racine N, Tough S (2020). Prevalence of preschoolers meeting vs exceeding screen time guidelines. JAMA Pediatr.

[44] Nathanson AI (2001). Parent and child perspectives on the presence and meaning of parental television mediation. J Broadcasting Electronic Media.

[45] European Food Safety Authority (2009). General principles for the collection of national food consumption data in the view of a pan-European dietary survey. EFSA J.

[46] Vanderloo LM, Carsley S, Aglipay M, Cost KT, Maguire J, Birken CS (2020). Applying harm reduction principles to address screen time in young children amidst the COVID-19 pandemic. J Dev Beh Ped.

[47] Jago R, Zahra J, Edwards MJ (2016). Managing the screen-viewing behaviours of children aged 5–6 years: a qualitative analysis of parental strategies. BMJ Open.

[48] Nathanson AI, Beyens I (2018). The role of sleep in the relation between young children's mobile media use and effortful control. Brit J Dev Psych.

